# Digital detection of Alzheimer’s disease using smiles and conversations with a chatbot

**DOI:** 10.1038/s41598-024-77220-0

**Published:** 2024-11-01

**Authors:** Haruka Takeshige-Amano, Genko Oyama, Mayuko Ogawa, Keiko Fusegi, Taiki Kambe, Kenta Shiina, Shin-ichi Ueno, Ayami Okuzumi, Taku Hatano, Yumiko Motoi, Ito Kawakami, Maya Ando, Sachiko Nakayama, Yoshinori Ishida, Shun Maei, Xiangxun Lu, Tomohisa Kobayashi, Rina Wooden, Susumu Ota, Ken Morito, Yoshitaka Ito, Yoshihiro Nakajima, Asako Yoritaka, Tadafumi Kato, Nobutaka Hattori

**Affiliations:** 1 Department of Neurology, Faculty of Medicine, Juntendo University, 2-1-1 Hongo, Bunkyo-Ku, Tokyo, 113-8421 Japan; 2 Department of Neurology, Faculty of Medicine, Juntendo University Koshigaya Hospital, Saitama, Japan; 3 Department of Psychiatry, Faculty of Medicine, Juntendo University, Tokyo, Japan; 4 Department of Neurology, Takeda Sogo Hospital, Fukushima, Japan; 5 IBM Consulting, IBM Japan, Ltd., Tokyo, Japan; 6 GLORY Ltd., Tokyo, Japan; 7 Neurodegenerative Disorders Collaborative Laboratory, RIKEN Center for Brain Science, Saitama, Japan

**Keywords:** Alzheimer's disease, Machine learning

## Abstract

In super-aged societies, dementia has become a critical issue, underscoring the urgent need for tools to assess cognitive status effectively in various sectors, including financial and business settings. Facial and speech features have been tried as cost-effective biomarkers of dementia including Alzheimer’s disease (AD). We aimed to establish an easy, automatic, and extensive screening tool for AD using a chatbot and artificial intelligence. Smile images and visual and auditory data of natural conversations with a chatbot from 99 healthy controls (HCs) and 93 individuals with AD or mild cognitive impairment due to AD (PwA) were analyzed using machine learning. A subset of 8 facial and 21 sound features successfully distinguished PwA from HCs, with a high area under the receiver operating characteristic curve of 0.94 ± 0.05. Another subset of 8 facial and 20 sound features predicted the cognitive test scores, with a mean absolute error as low as 5.78 ± 0.08. These results were superior to those obtained from face or auditory data alone or from conventional image depiction tasks. Thus, by combining spontaneous sound and facial data obtained through conversations with a chatbot, the proposed model can be put to practical use in real-life scenarios.

**Supplementary Information:**

The online version contains supplementary material available at 10.1038/s41598-024-77220-0.

## Introduction

People over 60 years old are projected to account for one-sixth of the global population by 2030, and their number will double by 2050^1^. The number of patients with dementia is increasing rapidly in super-aged societies, particularly in Japan. One in nine people over the age of 64 years is estimated to have cognitive decline^2^. The burden of dementia extends beyond healthcare, impacting various sectors of society, including financial and business settings. According to the Bank of Japan, more than 60% of household financial assets in Japan are held by individuals over the age of 60 years. Although their economic activities are vital, their financial capacity cannot be assessed based solely on their age. This underscores the urgent need for tools that can evaluate cognitive status effectively, anywhere, and by anyone, especially in financial or business contexts.

Alzheimer’s disease (AD) accounts for 50–75% of dementia cases. Facial expressions and speech are sometimes distinctive in patients with AD and thus may be valuable biomarkers for the development of a cost-effective AD screening method^2–6^. Facial features of AD include hypomimia, reduced facial emotion expressivity^7^, and changes in facial action units, eye movement, and lip activity^8,9^. AD patients also reportedly move their face and eyes in the same direction simultaneously^10^. Features of speech include temporal features, such as longer speech and phonation times, more pauses, a lower speech rate, a lower articulation rate, shorter-duration phrasal segments^11–16^, impaired emotional prosody^17^, and poorer speech signals^18^, whereas phonological features include changes in spectral features, such as mel-frequency cepstral coefficients (MFCCs)^19^, the amplitude of sound and the harmonics/noise ratio^16^, parameters f2 and f3^16^, formants^20^ and syllables^14^.

Recently, artificial intelligence (AI) has been attracting attention as a tool to discriminate patients with AD from healthy controls (HCs) on the basis of these features. AI analyses of facial features to detect AD or dementia have been reported in several papers (Supplementary Table 1)^2,5,6^. Efforts to establish acoustic measures to diagnose AD using AI began approximately a decade ago, with many more reports compared with the number on facial features^3^. Previous studies performed to detect AD using machine learning are detailed in Supplementary Table 2^13,15,19–31^ (adapted from reference^3^). There is also a recent study that used speech data to predict the progression from mild cognitive impairment (MCI) to AD within 6 years^32^. However, there are no studies that used both facial and auditory data. In AI analyses, machine learning algorithms make judgements on the basis of manually selected features, whereas deep learning algorithms learn the features by autonomously. Previous studies reporting high accuracy used deep learning, although this has a high risk of overfitting^3,5,6^. Additionally, most studies on auditory features used data obtained during cognitive tests and tasks, which are stressful for people with cognitive decline. Other studies used interviews with medical staff, which can only be performed in certain situations.

In our study, using machine learning, we aimed to develop an easy, automatic, and extensive tool to screen patients with AD that is enjoyable, i.e., does not induce stress. We analyzed smile images and acoustic and visual data extracted from videos of natural conversations with a chatbot and analyzed these data according to patients’ diagnoses and cognitive test scores.

## Methods

### Study design

In this cross-sectional study, individuals with AD or MCI due to AD (PwA) were recruited from among visitors to the outpatient clinic of the Neurology or Psychiatry Department of Juntendo University Hospital, Juntendo Koshigaya Hospital, or Takeda General Hospital from June 1, 2022, to December 31, 2022. The diagnoses of AD and MCI due to AD were made according to the National Institute on Aging and Alzheimer’s Association criteria^33^. HCs (i.e., those without any neurological diseases) were recruited by a recruitment company (https://3h-ct.co.jp/) during the same study period and matched to patients by age and sex. The sex of HCs was controlled by exact matching with PwA, and we restricted the age difference between matched PwA and HCs to within 5 years. The inclusion criteria for participation were as follows: (1) aged over 65 years; (2) native Japanese speaker; and (3) ability to use the application. The exclusion criteria were as follows: (1) speech problems so severe that speech was undetectable by a tablet microphone; and (2) inability to use the application. General information such as age, sex, and past medical history were obtained using the application.

This study consisted of two sessions: an application session, which comprised a cognitive test and a conversation with a chatbot, and a cognitive assessment session that comprised the Mini-Mental State Examination-Japanese (MMSE-J), the Hasegawa Dementia Scale-Revised (HDS-R), the Japanese version of the Montreal Cognitive Assessment (MoCA-J), the Trail Making Test (TMT), the Neuropsychiatric Inventory (NPI), and the Geriatric Depression Scale (GDS). The tests in the cognitive assessment session were administered by licensed psychologists.

The order of the application and cognitive assessment sessions was randomized to control for potential order effects. Both sessions were conducted in a quiet brightly lit room of the hospital during one visit, except in a few cases in which the patient was unable to complete the sessions in one day. Both PwA and HCs were tested in the same environment. The application section was completed by the participants themselves, who followed the staff’s instructions. Sample photos from the experiment are shown in Supplementary Fig. 1. All participants were using the application for the first time and were required to finish all of the tasks in one session. Participants who left midway during the experiment were excluded.

### Equipment and online application

An iOS mobile application was developed to collect data during the testing session. The application was built using the Amazon Web Service (AWS, Seatle, WA, USA), including the AWS lambda and the AWS Application Programming Interface (API) Gateway. Unstructured (i.e., video, figure, and sound) data were stored in the Simple Cloud Storage (S3) of the AWS using the WebRTC protocol (an open source-project for real-time communication in applications; https://webrtc.org/), and structured (i.e., text answers) data were stored in the AWS Relational Database Service. We used a facial expression recognition Application Programming Interface, supported by GLORY Ltd. (Tokyo, Japan).The iOS application was installed on an iPad device (Apple, Cupertino, CA, USA). The application consisted of three parts: a smile test session, a chatbot talk session, and a cognitive test session. Assuming that older adults may possess less technical knowledge and fewer digital skills, we implemented visually simple and intuitive designs with voice guidance. We also introduced elements such as speech bubble text displays to support further actions, waveform displays during voice recording, and tutorials for all measurements, enhancing visual and auditory aspects to provide an easily understandable interface. Tasks appear one at a time: the next task appears after finishing the previous one. Participants were asked to remove their surgical masks they were wearing to prevent COVID-19 infection, in order to properly assess their faces and voices during the application session. Sample images of the user interface are shown in Supplementary Fig. 2.

In the smile test session, the participant was provided with audio and visual instructions to smile and then return to a neutral face. For each instruction, facial expressions were captured for 15 s (smile test video). The smile test session was performed twice for each participant. If there were problems with the facial data (e.g., face out of frame), the smile test session was repeated once.

The application cognitive test included 35 questions. One additional external question taken from the Cookie Theft picture from the Boston Diagnostic Aphasia Examination Third Edition (BDAE-3) was also used.

Among the 35 cognitive questions, 11 had multiple-choice answers, which accounted for 15 points; 18 required audio responses, totaling 30 points; and 6 involved on-screen interactions, such as drawing and moving objects, which accounted for 6 points. These questions were formulated on the basis of the MMSE, MoCA-J, and TMT and spanned several cognitive domains: memory: 4 questions (12 points); language: 11 questions (11 points); attention: 4 questions (8 points); calculation: 1 question (1 point); executive function: 5 questions (9 points); and orientation: 10 questions (10 points). Of the 35 cognitive questions, 11 (15 points) were scored automatically, and the remaining 24 were scored manually by a psychologist. Detailed information on the 35 cognitive questions is provided in Supplementary Table 3.

The additional external question, which used the Cookie Theft picture from the Boston Diagnostic Aphasia Examination Third Edition, was designed to evaluate speech fluency and executive function. In the task, a drawing of a complicated kitchen scene is shown, in which a woman is wiping dishes, and two children are trying to obtain cookies from a cupboard. Auditory data from an image-description task including this picture have been used in previous studies to discriminate patients with AD from HCs^13,19,25,28,29,34^, partially because there is an accessible databank that includes these data. In the present study, this stimulus appeared on the participants’ tablets, and they were asked to orally describe it. Responses were recorded for up to 5 min and transformed into audio features, which were compared with the features generated from the chatbot questions. Details of the external question are provided in Supplementary Table 4. At the end of the application session, the total score and standard deviation were provided as feedback to the participants.

The chatbot session included seven questions that were inserted before and after the cognitive testing session. The chatbot asked general questions, for example about the participants’ hobbies, favorite foods, and impressions of the cognitive test. All questions in the chatbot session were answered orally, and both audio and facial expression data were recorded (chatbot talk video). The maximum answer time (response time), which was controlled by the app, was 5 min for each chatbot question. After finishing a question, the participants pressed the “next” button to move to the next one. Psychologists reminded the participants to press the button. The questions used in the cognitive test and chatbot sessions are listed in Supplementary Tables 3–5 in the supplementary materials. The data collection, processing, and analysis steps are shown in Supplementary Fig. 3.

### Video data and facial expression features

As described in the previous section, two types of videos were recorded: a smile test video and a chatbot talk video. The recording conditions were as follows: resolution, 1,280 × 960 pixels; frame rate, 30 fps; and compression format H.264 (approximately 2,300 kbps).

We used the smile index^35^ to evaluate the facial features in the smile test video. The processing of the smile index consists of two steps. First, the system extracts a facial area from the image using a deep learning-based model trained on a general facial image dataset, primarily depicting healthy people. Next, the system calculates the smile index from the aforementioned facial image using another deep learning-based model trained on a dataset containing thousands of smiling and neutral faces, mostly of healthy people. In particular, the latter model is trained to output the degree of smiling corresponding to the input face image, and the smile index obtained as the output takes scalar values ranging from 0 (neutral) to 100 (smiling).

A smiling section of video was defined as a section with a relatively high smile index. The boundaries of the smiling section were determined using a method that identifies a region in which there is a significant change in the smile state, as indicated by the difference between local smile indexes.

**Fig. 1:**
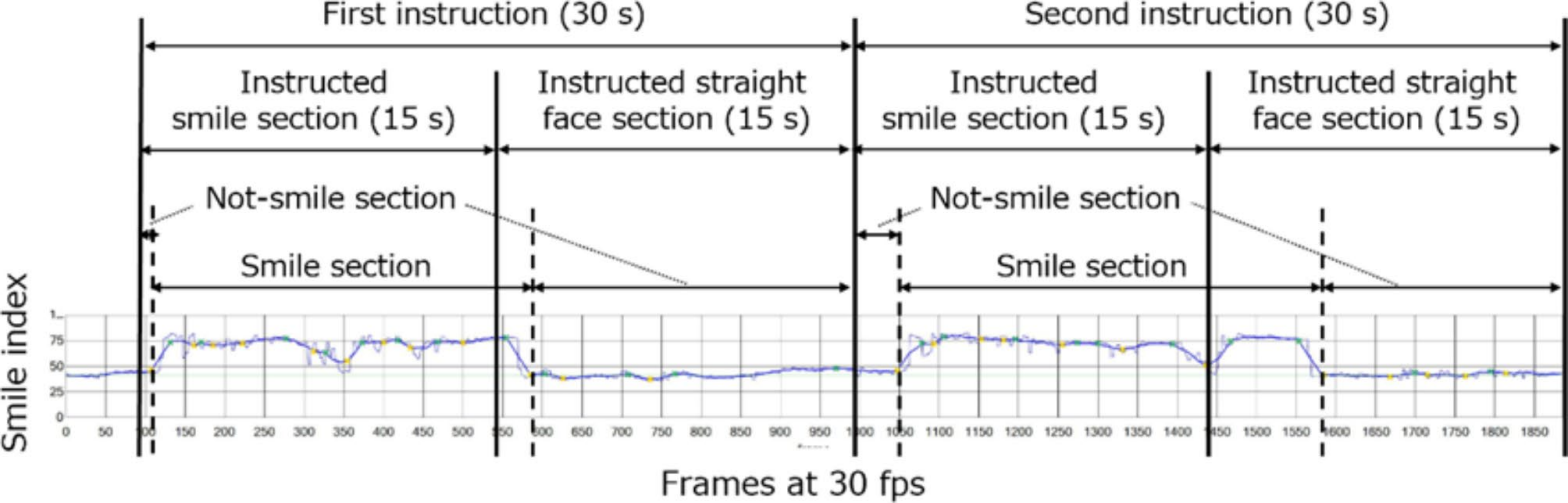
Composition of sections considered when extracting of facial features from the smile test.

Sections in which participants were instructed to smile were designated as instructed smiling sections, and sections in which participants were instructed to make a straight face were designated as instructed straight-face sections (Fig. 1). Participants with fewer than two adequate-quality videos were excluded from the analysis.

We used two videos per participant to calculate the smile index using four types of smile-related features: (1) duration of smiling face in the smiling section; (2) time taken to smile following the instruction in the smiling section, indicated by the rising angle of the smile index; (3) amplitude and fluctuation of the smile index in the smiling and instructed smiling sections, calculated from the average, maximum, minimum, and standard deviation values of the smile index; and (4) difference in the strength of the smile index between smiling and non-smiling sections and between instructed smiling and instructed straight-face sections.

To evaluate the facial features in the chatbot talk video, we used the smile index as detailed above, as well as the orientation index, which indicates the angles (elevation, azimuth, and rotation) of the face; the eye-opening index, which indicates the degree of eyes opening; and the blink index, which denotes the blink behavior estimated from the eye-opening index. A total of 48 facial features were extracted from the video data for further analyses according to previous reports and the experiences of neurologists.

### Audio data and sound features

The audio data were recorded at a 12-kHz sampling rate in MPEG-4 audio format. To prevent effects of background noise, we applied sound overlap processing to eliminate the noise. First, all audio files were manually checked, and the start and end times of the overlapping sections were tagged. Second, the overlapping sound sections were wiped according to the tagged times and saved as a new sound file. Finally, all sound features were extracted from the new audio data.

We previously defined three types of sound features: linguistic, prosodic, and acoustic features^35^. Linguistic features are related to the lexical content of speech and include the statistics of filled pauses, parts of speech, and vocabulary richness. Prosodic features are related to the pitch, speech rate, and phonation time. Acoustic features are related to the statistics of formants (f0, f1, and f2), shimmers (variations in sound wave amplitudes), jitter (variations in sound wave frequencies), and MFCCs. In this study, audio answer data were saved for individual questions. To average the responses in the chatbot session, we combined the audio answer data from the entire chatbot session. All sound features were calculated from these combined data and processed using open-source library packages (Librosa = 0.8.1, https://github.com/librosa/librosa; Pydub = 0.25.1, https://github.com/jiaaro/pydub; Parselmouth = 0.4.3, https://github.com/YannickJadoul/Parselmouth; and Spacy = 3.4.4, https://github.com/explosion/spaCy). For the assessments, a total of 110 sound features, comprising 53 linguistic features, 6 prosodic features, and 51 acoustic features, were extracted from the audio data as suggested by previous reports and on an empirical basis according to neurology specialists (H. T-A., G.O., N.H.).

### Statistical analysis

We applied the Mann-Whitney U test to evaluate the differences in sound and facial expression features between PwA and HCs. Significant (*P* ≤ 0.01) features were then used for classification model building.

We applied Pearson’s correlation test to examine the relationships between features and the application cognitive test score. Features with a correlation of 0.1 or higher were used in the regression model building.

The statistical analysis and model building were performed using open-source library packages (scipy version 1.7.1 and scikit-learn version 1.0.1) in Python version 3.8.

## Results

### Participant characteristics

We screened a total of 209 participants, comprising 105 individuals with PwA and 104 HC participants who were considered to have no neurological diseases. Of these participants, 104 PwA and 104 HCs participated and completed the cognitive tests.

Eight participants were removed because of possible depression, i.e., when the GDS test score was ≥ 10, or because the GDS score was unavailable. Additionally, two participants with facial paralysis were removed, and another six participants’ data were unusable because of inadequate recording quality for the audio or video data (e.g., the recording was made while wearing a mask). Finally, 192 participants with full data available (93 PwA and 99 HCs) were included in the analysis. The demographic data of participants are shown in Table 1.

**Table 1:** Demographic data of the participants.

	HC	PwA	*p*-value
Number	99	93	-
Age [years], mean ± SD	78.4 ± 4.5	79.9 ± 5.1	0.03
Sex (Male/Female), mean ± SD	49/50	42/51	-
App cognitive test, mean ± SD	38.9 ± 5.6	24.3 ± 9.1	< 0.01
MMSE-J, mean ± SD	27.6 ± 1.6	20.8 ± 4.9	< 0.01
MoCA-J, mean ± SD	25.7 ± 2.5	16.5 ± 5.2	< 0.01
HDS-R, mean ± SD	28.5 ± 1.6	20.2 ± 5.9	< 0.01
TMT-A [sec], mean ± SD	49.6 ± 22.1	90.3 ± 47.8	< 0.01
TMT-B [sec], mean ± SD	102.3 ± 46.5	211.6 ± 83.2	< 0.01
GDS, mean ± SD	1.9 ± 2.0	2.8 ± 2.3	< 0.01

Abbreviations: MMSE-J, Mini-mental State Examination-Japanese; MoCA-J, Japanese version of the Montreal Cognitive Assessment; HDS-R, Hasegawa Dementia Scale-Revised; TMT, Trail Making Test; GDS, Geriatric Depression Scale, HC; healthy control, PwA; persons with Alzheimer’s disease or mild cognitive impairment due to Alzheimer’s disease.

### Validation

#### Validity

In the current study, cognitive tests were conducted in two sessions: the application cognitive test, comprised of tasks extracted from the MMSE, HDS-R, MoCa-J and TMT, and conventional tests were conducted in a second session by licensed psychologists. The application cognitive test is simpler and can be executed without medical staff. The AI analysis of smile and chatbot data aimed to estimate the application cognitive test score for future studies. Before using this score, its validity and reliability were examined in conventional tests by psychologists. Validity was evaluated across two sessions: the first session involved a mobile application-based cognitive test, and the second session involved cognitive assessments, including the MMSE-J, HDS-R, MoCA-J, TMT, NPI, and GDS. These assessments were conducted by licensed psychologists.

We applied the Pearson correlation test to assess the relationships between the mobile application cognitive test results and the MMSE-J, HDS-R, MoCa-J, and TMT scores.

The NPI data were not used in this study because they were only acquired from the PwA group. In addition, NPI data were available for only 75 participants who were accompanied by their caregivers.

The mobile application cognitive test score strongly correlated with the MMSE, HDS-R, and MoCa-J scores, as well as the times required to complete the TMT-A and TMT-B (Supplementary Table 6). Therefore, our mobile application cognitive test had high validity.

#### Reliability

Reliability refers to the consistency and stability of test scores over time and across different testing conditions. We evaluated internal consistency using Cronbach’s alpha, which was 0.91, indicating good internal consistency and reliability of the mobile application cognitive test. We also confirmed the internal correlation of the test between the score of a single question and the total score of the remaining questions. The internal correlation coefficients ranged from 0.59 to 0.24 (*P* < 0.01) for all questions except Question 2: “Where are we now? Which city /town?” Question 2 was thus excluded because the participants may not have been familiar with the location of the hospital. These results showed that the mobile application cognitive test had adequate internal consistency and reliability.

### PwA versus HCs

#### Confounding test

We applied the confounding test to identify confounding variables, that is, factors that can influence both the independent (i.e., sound and facial features) and dependent variables (i.e., dementia stage) and could lead to inaccurate relationships between the variables of interest. We evaluated the following potentially confounding variables:

- demographic factors: age and sex.
- socioeconomic factors: education.

From the results in Supplementary Fig. 4, we can conclude that age, sex, and education were not confounding factors in our dataset. Therefore, we used all data in the statistical analysis and model building.

#### Significant facial and sound features between PwA and HCs

The Mann-Whitney U test was used to evaluate differences in sound and facial expression features between PwA and HCs. Of the 158 features, 81 (comprising 19 facial and 62 sound features) showed a significant difference between the groups (*P* ≤ 0.01). The complete results of the Mann-Whitney U test are provided in Supplementary Table 7.

The significant sound features included prosodic features, such as the MFCCs and f2, and linguistic features, such as silence time and the Brunet index of vocabulary. The above-mentioned features all had medium-to-large effect sizes for the differences between PwA and HCs. In the correlation analysis between these features and cognitive decline, the frequency of particles and nouns, number of words, and the Brunet index of vocabulary showed a large effect size, indicating a reduction in lexical richness.

Significant differences were observed for facial features between the smiling and non-smiling sections. The smile features, including the difference in average and maximum smile values between the smiling and non-smiling sections, had a medium effect size when comparing with the PwA and HCs. This difference showed a positive correlation with the mobile application cognitive test score, which indicated reduced smile expression in PwA. The 81 features that showed a significant group difference (*p* < 0.01) were used to build the classification model for differentiating PwA from HCs.

#### Classification model

We developed classification models for PwA and HC using a combination of audio and facial features. We selected features that were significant (*P* ≤ 0.01) in the Mann-Whitney U test. In addition, to account for collinearity, features with a correlation of 0.5 or higher with other features were excluded. Finally, 29 features, including 8 facial features and 21 sound features, were used for model predictions.

Data splitting was performed using 10-fold cross-validation. Performance metrics included the area under the receiver operating characteristic curve, precision, recall, accuracy, and specificity (Table 2). We compared the performance of the linear (i.e., logistic regression) and nonlinear (random forest, XGBoost [eXtreme Gradient Boosting]^36^, and Light GBM [Gradient Boosting Machine]) models^37^.

**Table 2:** Model performance using different combinations of machine learning algorithms (random forest, logistic regression, XGBoost, and light GBM) and feature sets (facial expression only, sound only, and both) for PwA classification.

No	Algorithm	Feature set	AUC	Accuracy	Precision	Recall	Specificity
1	Random Forest	only facial	0.76 ± 0.11	0.72 ± 0.10	0.72 ± 0.13	0.72 ± 0.16	0.72 ± 0.16
2		only sound	0.92 ± 0.06	0.85 ± 0.07	0.85 ± 0.09	0.85 ± 0.11	0.85 ± 0.10
3		facial + sound	0.94 ± 0.05	0.86 ± 0.08	0.88 ± 0.10	0.84 ± 0.11	0.88 ± 0.12
4	Logistic Regression	only facial	0.78 ± 0.11	0.70 ± 0.10	0.70 ± 0.11	0.71 ± 0.14	0.70 ± 0.14
5		only sound	0.86 ± 0.07	0.78 ± 0.08	0.78 ± 0.11	0.78 ± 0.13	0.78 ± 0.13
6		facial + sound	0.94 ± 0.05	0.85 ± 0.08	0.85 ± 0.10	0.86 ± 0.11	0.85 ± 0.11
7	XGBoost	only facial	0.74 ± 0.1	0.67 ± 0.10	0.67 ± 0.12	0.65 ± 0.16	0.69 ± 0.13
8		only sound	0.91 ± 0.07	0.83 ± 0.07	0.84 ± 0.09	0.83 ± 0.11	0.84 ± 0.10
9		facial + sound	0.93 ± 0.05	0.85 ± 0.08	0.86 ± 0.10	0.84 ± 0.13	0.86 ± 0.12
10	Light GBM	only facial	0.73 ± 0.11	0.68 ± 0.10	0.68 ± 0.12	0.67 ± 0.14	0.69 ± 0.15
11		only sound	0.84 ± 0.10	0.83 ± 0.09	0.83 ± 0.11	0.84 ± 0.13	0.82 ± 0.13
12		facial + sound	0.93 ± 0.05	0.85 ± 0.08	0.85 ± 0.10	0.84 ± 0.12	0.85 ± 0.12

Abbreviations: XGBoost, eXtreme Gradient Boosting; GBM, Gradient Boosting Machine; PwA, persons with Alzheimer’s disease or mild cognitive impairment due to Alzheimer’s disease; AUC, area under the receiver operating characteristic curve.

**Fig. 2:**
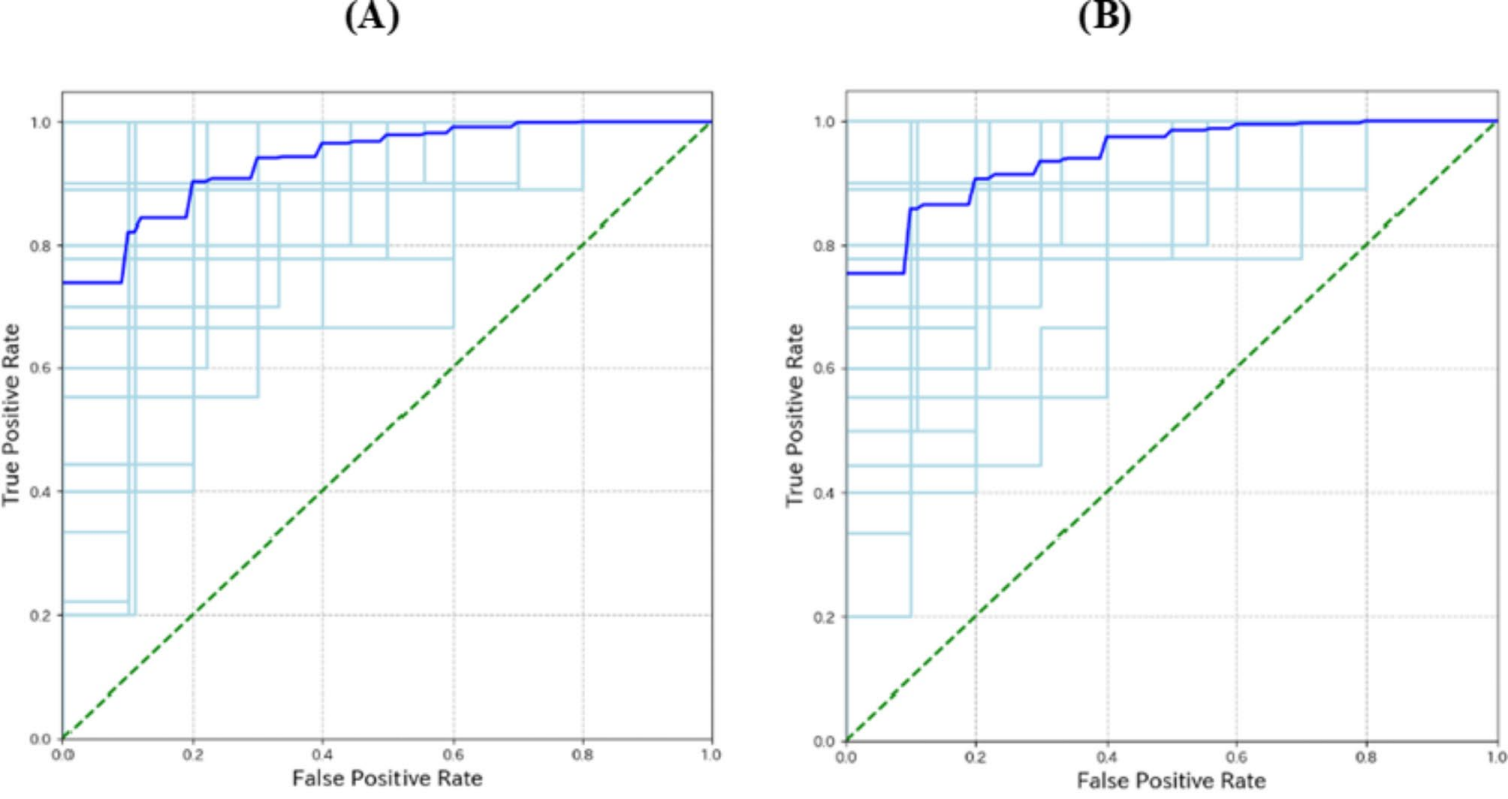
Receiver operating characteristic (ROC) curve of the machine learning algorithms. (**A**) Random forest algorithm using facial and sound feature sets (No. 3 in Table 2). (**B**) Logistic regression algorithm using facial and sound feature sets (No. 6 in Table 2). These algorithms had the best classification performance. The horizontal axis is the false-positive rate, and the vertical axis is the true-positive rate. The light blue lines are the ROC curves of the cross-validation, and the dark blue lines are the averaged ROC curves. The green dashed line indicates the performance of a random classifier.

The random forest and logistic regression algorithms using the facial and sound features showed the best classification performance. The receiver operating characteristic (ROC) curves of the random forest and logistic regression models are shown in Fig. 2.

### Cognitive score study

#### Regression model

We built regression models to predict the mobile application cognitive test scores from the sound and/or face features in all participants. Features with a correlation coefficient of ≥ 0.1 with the mobile application cognitive test score were selected to build the regression model. To account for collinearity, features with a correlation coefficient of 0.5 or higher with other features were excluded. In total, 28 features, including 8 facial features and 20 sound features, were used for model prediction.

**Fig. 3:**
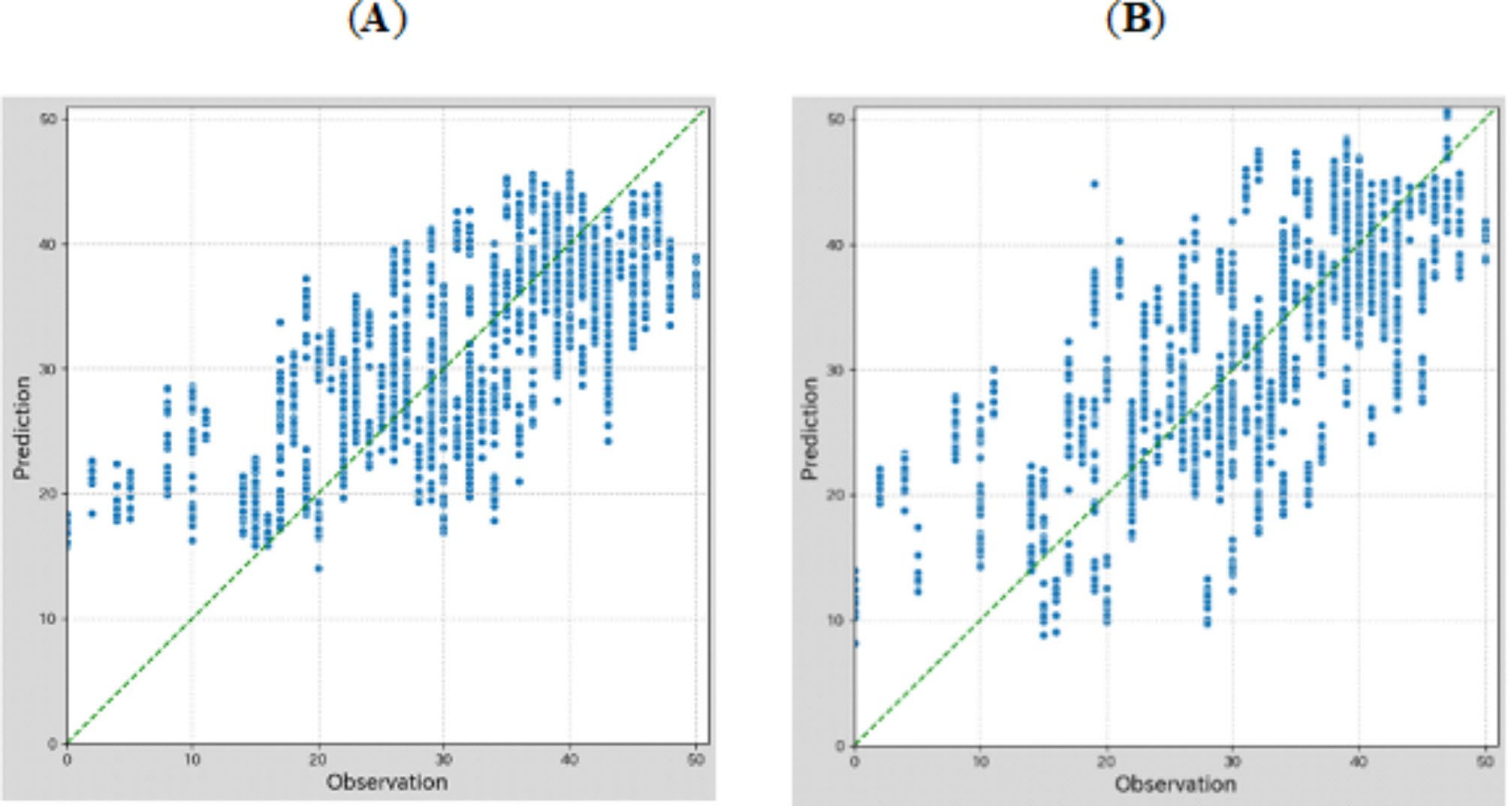
Distribution of the mobile application cognitive test score prediction using different algorithms. (**A**) light gradient boosting machine algorithm with facial and sound feature sets (No. 12 in Table 3). (**B**) Ridge regression algorithm with facial and sound feature sets (No. 6 in Table 3). The horizontal axis (observation) is the true application cognitive test scores, and the vertical axis (prediction) is the predicted application cognitive test scores. The blue lines show the correlation between the true and predicted values, and the green dashed lines indicate the score distribution when the correlation coefficient = 1.

Data splitting was performed using 10-fold cross-validation, and the regression performance was evaluated using the mean absolute error (MAE) and the correlation coefficient between the predicted and actual values. We compared the regression results among different feature groups and algorithms. The model performance results are shown in Table 3. The Light GBM and ridge regression algorithms using a combination of the sound and facial features had the best the mobile application cognitive test score prediction performance. The distributions of the cognitive test score predictions are shown in Fig. 3.

**Table 3:** Model performance using different combinations of machine learning algorithms (random forest, ridge regression, XGBoost, and light GBM) and feature sets (facial expression only, sound only, and both) for cognitive score prediction (regression).

No	Base Algorithm	Feature set	Correlation	MAE
1	Random Forest	only facial	0.44 ± 0.01	7.45 ± 0.07
2		only sound	0.70 ± 0.01	6.01 ± 0.05
3		facial + sound	0.71 ± 0.01	5.82 ± 0.05
4	Ridge Regression	only facial	0.49 ± 0.01	7.30 ± 0.03
5		only sound	0.69 ± 0.00	5.90 ± 0.09
6		facial + sound	0.71 ± 0.01	5.78 ± 0.08
7	XGBoost	only facial	0.39 ± 0.02	7.82 ± 0.11
8		only sound	0.66 ± 0.01	6.24 ± 0.10
9		facial + sound	0.67 ± 0.01	6.05 ± 0.09
10	Light GBM	only facial	0.40 ± 0.01	7.58 ± 0.03
11		only sound	0.70 ± 0.01	5.81 ± 0.09
12		facial + sound	0.71 ± 0.01	5.71 ± 0.09

Abbreviations: XGBoost, eXtreme Gradient Boosting; GBM, Gradient Boosting Machine; MAE, mean absolute error.

#### Correlation test

We applied Spearman’s correlation test to examine correlations between the mobile application cognitive test score and all 158 features. Of the 158 features, 19 facial features and 60 sound features were positively or negatively correlated (|r| ≥ 0.2) with cognitive test scores across the entire dataset. When we analyzed the groups separately, 2 facial features and 37 sound features were positively or negatively correlated (|r| ≥ 0.2) with cognitive test scores in the HC group. In contrast, 6 facial features and 22 sound features were positively or negatively correlated with the cognitive test score in the PwA group. This suggests that the feature values of HCs and PwA have distinct characteristics. The complete results of the correlation test are provided in Supplementary Tables 8–10, and detailed feature definitions are listed in Supplementary Table 11.

In the HC group, for facial features, the average smile score was strongly positively correlated with the mobile application cognitive test score. In the PwA group, the standard deviation of facial movement was strongly negatively correlated with the mobile application cognitive test score. For the sound features, in the HC group, the mobile application cognitive test score was significantly positively correlated with the frequency of adposition, verb, and adjective use and significantly negatively correlated with the proportion of silence time. In the PwA group, the mobile application cognitive test score was significantly positively correlated with the frequency of adjective use and negatively correlated with the use of pronouns.

### Cookie theft question

We also evaluated the sound features derived from both chatbot questions and the Cookie Theft question. Using the same data-splitting and feature-engineering method used for the previous models, we assessed the performance of both the classification and regression models. Our analysis revealed that the models using sound features from the chatbot questions performed as well as, or better than, those using features from the Cookie Theft question. The complete model performance results are shown in Supplementary Table 12.

### t-distributed stochastic neighbor embedding (t-SNE) analysis

We performed t-SNE analysis to obtain an overview of the features. The results are shown in Supplementary Tables 13 and Supplementary Fig. 5. Dimension 1 had the highest correlation with facial expression features, and dimension 2 had the highest correlation with speech features. In dimension 1, there was a tendency for the values of HCs to be small, and for the variation in the values of the patients to be large. Similarly, in dimension 2, the values for patients tended to be small, and the values for healthy participants tended to have a wide range.

## Discussion

We developed a robust method for distinguishing between PwA and age- and sex-matched HCs using only smiles and casual conversations with a chatbot, analyzed via machine learning. This method successfully detected PwA, shown by the high area under the ROC curve value of 0.94 ± 0.05, and it also estimated cognitive test scores with high accuracy, with a low MAE of 5.78 ± 0.08. Our study is notable in detecting PwA and estimating cognitive test scores with high accuracy by combining visual and auditory features, whereas previous reports only focused on one feature type. Analyses using combined features were superior to those using either visual or auditory features in the current study. Additionally, the t-SNE plot revealed that the dimensions characterizing participants differed between the speech and facial expression features, suggesting that combining these features may be more effective for detecting cognitive functions. Another strength of our study is the usage of machine learning: previous studies reporting high accuracy tended to use deep learning, which has a higher risk of overfitting^3,5,6^ (Supplementary Table 1).

We introduced an innovative approach for evaluating cognitive status using a tablet application that did not induce stress in patients. Our screening tablet application asked examinees to simply smile and have a casual conversation with a chatbot. This constitutes a simple, automatic, and highly versatile screening approach without the need for human intervention or time-consuming and costly examinations, such as magnetic resonance imaging, single-photon emission computed tomography, or demanding cognitive assessments. Previous studies aiming to detect AD using sound features captured auditory data from cognitive test tasks, such as reading or repeating tasks, or from the Cookie theft question, in which participants describe the scene in a particular picture. Our comparison between the Cookie Theft question and chatbot questions showed that the models using the sound features from the chatbot questions performed as well as, or better than, those using features from the Cookie Theft question, which suggests that free-conversation (i.e., chatbot) questions are more effective at capturing cognition-related sound features than is the structured Cookie Theft question. Our study is noteworthy in this regard, pointing to the possibility of flexibility in crafting questions for specific cases.

Furthermore, to the best of our knowledge, no study on the facial expressions and sounds of PwA have examined whether age or sex are confounding factors, even though facial and sound features are greatly influenced by these factors. The use of data matching strengthens the robustness of our study findings.

The features that were significantly correlated with app cognitive test scores in this study were informative. For instance, the smile score was strongly correlated with the app cognitive test score in HCs. Strong engagement in social and mental activities in later life reportedly protects against dementia^38^. It has been shown that older people with larger social networks have better cognitive status regardless of their pathological status^39^. The higher smile scores in our study may indicate greater mental and social activity of the participants, which could result in good cognitive status. In PwA, the standard deviation for facial movement negatively correlated with the app cognitive test score. This may reflect the “head-turning sign” or “saving appearances responses”, which are characteristic of AD.

The recent development of disease-modifying therapy has made early diagnosis of AD more important than it has been in the past. This new tool could contribute greatly to screening for AD and referring patients to medical institutions early in the course of the disease. Furthermore, consumer issues involving individuals with AD have become increasingly serious problems. It can be difficult for businesspersons to notice cognitive decline in their clients because politeness is usually retained in the early stages of AD. The tool we assessed in this study could aid screening for cognitive impairment in older clients in business settings, thus facilitating an active and safe society for everyone.

Our study has several limitations relating to the acquisition of data and the model algorithms used. Regarding the acquisition of data, our sample was limited to individuals aged over 65 years. The PwA were diagnosed according to clinical diagnostic criteria, without confirmation via biomarker or pathology analysis. However, all patients were diagnosed by specialists. Although, the HCs self-declared their cognitive status, none of the participants had significant cognitive impairment (i.e., scored below 21 points on the HDS-R). For both the PwA and HC groups, we recruited only those who were able to use a tablet. Thus, severe PwA cases were not included. However, because our aim was to establish a screening tool, we considered it most appropriate to focus on mild-to-moderate PwA. Notably, many older Japanese adults are reluctant to use tablets and computers, which may have impacted our results. Regarding the model algorithms used, although ensemble models, such as random forest, XGBoost, and Light GBM, achieved the highest accuracy, they also present a risk of overfitting. In contrast, logistic regression and ridge regression models have a lower risk of overfitting, and we found that these models had slightly lower accuracy; therefore, the latter models may have better generalizability.

The utility of spontaneous sound and facial data offers practical applications for the proposed models in real-life scenarios. Future studies could explore additional features and modalities to enhance the performance of the screening tool.

## Electronic supplementary material

Below is the link to the electronic supplementary material.

Supplementary Material 1

## Data Availability

Data is provided within the manuscript or supplementary information files. Anonymized data will be shared with qualified researchers upon request to the corresponding authors (GO and NH).
